# Modular Control of Human Movement During Running: An Open Access Data Set

**DOI:** 10.3389/fphys.2018.01509

**Published:** 2018-10-29

**Authors:** Alessandro Santuz, Antonis Ekizos, Lars Janshen, Falk Mersmann, Sebastian Bohm, Vasilios Baltzopoulos, Adamantios Arampatzis

**Affiliations:** ^1^Department of Training and Movement Sciences, Humboldt-Universität zu Berlin, Berlin, Germany; ^2^Berlin School of Movement Science, Humboldt-Universität zu Berlin, Berlin, Germany; ^3^Research Institute for Sport and Exercise Sciences, Liverpool John Moores University, Liverpool, United Kingdom

**Keywords:** muscle synergies, locomotion, running, motor control, EMG, data set

## Abstract

The human body is an outstandingly complex machine including around 1000 muscles and joints acting synergistically. Yet, the coordination of the enormous amount of degrees of freedom needed for movement is mastered by our one brain and spinal cord. The idea that some synergistic neural components of movement exist was already suggested at the beginning of the 20th century. Since then, it has been widely accepted that the central nervous system might simplify the production of movement by avoiding the control of each muscle individually. Instead, it might be controlling muscles in common patterns that have been called muscle synergies. Only with the advent of modern computational methods and hardware it has been possible to numerically extract synergies from electromyography (EMG) signals. However, typical experimental setups do not include a big number of individuals, with common sample sizes of 5 to 20 participants. With this study, we make publicly available a set of EMG activities recorded during treadmill running from the right lower limb of 135 healthy and young adults (78 males and 57 females). Moreover, we include in this open access data set the code used to extract synergies from EMG data using non-negative matrix factorization (NMF) and the relative outcomes. Muscle synergies, containing the time-invariant muscle weightings (motor modules) and the time-dependent activation coefficients (motor primitives), were extracted from 13 ipsilateral EMG activities using NMF. Four synergies were enough to describe as many gait cycle phases during running: weight acceptance, propulsion, early swing, and late swing. We foresee many possible applications of our data that we can summarize in three key points. First, it can be a prime source for broadening the representation of human motor control due to the big sample size. Second, it could serve as a benchmark for scientists from multiple disciplines such as musculoskeletal modeling, robotics, clinical neuroscience, sport science, etc. Third, the data set could be used both to train students or to support established scientists in the perfection of current muscle synergies extraction methods. All the data is available at Zenodo (doi: 10.5281/zenodo.1254380).

## Introduction

The popularity of endurance running has greatly increased over the last few decades (Burfoot, 2007). Other than a purely social phenomenon, endurance running has been the center of attention in many research fields. Evolutionary anthropology, for instance, has been used to try to explain why humans show exceptional endurance running speeds compared to non-human primates (Bramble and Lieberman, 2004). In the neurosciences, running has long been ideal object for the investigation of movement due to its automatized, synergistic, general, cyclic, and phylogenetically old nature (Bernstein, 1967). However, a consensus as to how humans coordinate this repetitive and highly stereotyped movement is still far from being reached, mostly because direct experimental proofs are lacking.

Since the second half of last century it has been suggested that the central nervous system might simplify the production of movements by avoiding the complexity of activating each muscle individually (Bernstein, 1967; Lee, 1984; Bizzi et al., 1991, 2008). This feature might be implemented by linearly combining a small set of time-dependent commands, which have been called muscle synergies (Tresch et al., 1999). The idea that some synergistic neural components of movement exist was already suggested by Sherrington (1906) at the beginning of the 20th century. However, the so-called degrees of freedom problem and related principle of motor abundance was formally described only a few decades later (Bernstein, 1967). Following the thoughts of Bernstein, Lee (1984) published an essay in which the idea of “neuromotor synergies,” defined as neurally based units of voluntary action, was explored and supported. Bizzi et al. (1991) were the first to experimentally show spinal synergies, which they represented as force fields. However, only in 1999 Lee’s ideas could be numerically represented by showing the movement-specific recruitment of a small set of synergistic muscles in the spinal frog (Tresch et al., 1999). In the same year, Lee and Seung (1999) introduced the non-negative matrix factorization (NMF), a computational tool for extracting synergies from any kind of analyzed variable. To date, the NMF is one of the most common methods for reducing the high dimensional electromyographic (EMG) input into a small number of synergies (D’Avella, 2016).

Compared to the direct analysis of EMG signals, the muscle synergies concept has the clear advantage of being a high-throughput approach for analyzing muscle activities. In fact, it does not only provide the researcher with an automatic, low-dimensional, clustering of the activations during the gait cycle, but it also identifies the weighted contribution of each muscle for producing a certain movement. A number of studies were able to provide indirect evidence that synergies reside in the brainstem or spinal cord and follow a modular organization (Tresch et al., 2002; Hart and Giszter, 2004; Bizzi et al., 2008; Roh et al., 2011; Bizzi and Cheung, 2013). Synergies can be seen as low dimensional units that, via descending or afferent pathways, produce a complex electromyographic (EMG) pattern in muscles (Tresch et al., 2002; Bizzi and Cheung, 2013), creating a locomotor drive mediated by a certain amount of supraspinal control (Overduin et al., 2015). Synergies similar to those found in humans at a spinal (Ivanenko et al., 2006) or muscular level can be observed also in the motor cortex of the primate (Overduin et al., 2015) and cat (Yakovenko et al., 2011). This suggests a high degree of cooperation within the central nervous system’s structure at all levels.

Amongst the various types of locomotion, running has been object of several studies involving muscle synergies (Cappellini et al., 2006; Lacquaniti et al., 2012; Hagio et al., 2015; Yokoyama et al., 2016; Nishida et al., 2017; Santuz et al., 2017a,b, 2018). It is well accepted that human running can be described, in young and healthy individuals, with a few muscle synergies (Cappellini et al., 2006; Santuz et al., 2017b, 2018). Specifically, when analyzing the EMG activities of lower limb muscles, usually 4 or 5 synergies are observed (Cappellini et al., 2006; Santuz et al., 2017b, 2018). However, the data sets considered by existing studies are usually quite small (commonly including between 5 and 20 participants) and not freely available (Cappellini et al., 2006; Hagio et al., 2015; Yokoyama et al., 2016; Nishida et al., 2017; Santuz et al., 2017a,b, 2018). Moreover, a consensus regarding factorization techniques, data conditioning, and interpretation is not unanimous (Devarajan and Cheung, 2014; Oliveira et al., 2014; Santuz et al., 2017a; Shuman et al., 2017; Kieliba et al., 2018; Soomro et al., 2018). Human data sets are more and more frequently being published and made available to everyone (Makihara et al., 2012; Van Den Bogert et al., 2013; Wang and Srinivasan, 2014; Auton et al., 2015; Moore et al., 2015; Santos and Duarte, 2016; Fukuchi et al., 2018). However, sample sizes can be highly variable. The advantages related to the increased volume and variety of data sources mainly lie in the broadened representation of human variability, improvement of analysis strategies, and shareability for both scientific and educational purposes.

With this study, we present an open access data set of EMG and synergy data for running in young and healthy humans. The presented data is available in three formats: (1) the raw EMG, unprocessed together with the touchdown and lift-off timings of the recorded limb; (2) the filtered and time-normalized EMG; and (3) the muscle synergies extracted via NMF. Several applications based on this data set can be foreseen. From obtaining a deeper, more extended representation of motor coordination during running, to increase the detail of musculoskeletal models and robotic controls, passing through the improvement of current factorization methods and didactic purposes.

## Materials and Methods

### Experimental Protocol

For the development of the data set we recruited 135 volunteers (78 males and 57 females, height 175 ± 9 cm, body mass 69 ± 11 kg, age 30 ± 5 years, means ± standard deviation). The metadata file “participants_data.dat” includes the age and anthropometric data of the participants. All volunteers were regularly active and did not use orthotic insoles. None had any history of neuromuscular or musculoskeletal impairments, or any head or spine injury at the time of the measurements or in the previous 6 months. This study was reviewed and approved by the Ethics Committee of the Humboldt-Universität zu Berlin. All the participants gave written informed consent for the experimental procedure, in accordance with the Declaration of Helsinki.

The data recordings were performed while the participants were running on a treadmill (mercury, H-p-cosmos Sports & Medical GmbH, Nussdorf, Germany) equipped with a pressure plate recording the plantar pressure distribution at 120 Hz (FDM-THM-S, zebris Medical GmbH, Isny im Allgäu, Germany). The muscle activity of 13 ipsilateral muscles was recorded with a frequency of 1000 Hz using a 16-channel wireless bipolar EMG system (myon m320, myon AG, Schwarzenberg, Switzerland). For the EMG recordings, we used wet-gel silver/silver chloride electrodes with foam backing material and snap connector (BlueSensor N-00-S/25, Ambu A/S, Ballerup, Denmark).

After a self-selected warm-up (Santuz et al., 2017b), the participants ran on the treadmill at an average speed of 2.65 ± 0.31 m/s (details on speed are provided in the metadata file “participants_data.dat”) for the time necessary to record two trials of 60 s each. The reason for choosing this particular speed is that some participants ran at a pre-defined speed (2.2, 2.5, 2.8, or 3.0 m/s), while others were asked to find and run at their comfortable speed, depending on the experimental setup in which the data was collected (details on speed type are provided in the metadata file “participants_data.dat”). This was due to the fact that data was collected in different experimental setups. The procedure to find the comfortable speed was implemented using the method of limits (Treutwein, 1995), as follows. The speed was randomly increased with steps of 0.02 to 0.05 m/s at varying time intervals (around 5 to 10 s) until the participant was comfortable with a specific pace. The operation was then repeated starting from a faster speed and randomly decreasing it as previously done. If the comfortable speed value did not differ more than 10% from the previous, the average of the two values was taken as the preferred. Otherwise, the whole procedure was repeated as needed. In both the pre-defined and the preferred speed protocols, the warm-up procedure, including the selection of speed where applicable, typically lasted between 5 and 10 min.

### Gait Cycle Parameters

The pressure plate’s raw data was acquired using the proprietary software (WinFDM-T v2.5.1, zebris Medical GmbH, Isny im Allgäu, Germany) and then extracted in raw format for autonomous post-processing of the gait spatiotemporal parameters using a validated custom algorithm (Santuz et al., 2016) written in R version 3.5.1 (R Foundation for Statistical Computing, R Core Team, Vienna, Austria). As an indication of the foot strike pattern, the strike index was calculated using a validated algorithm based on the numerical analysis of foot pressure distribution (Santuz et al., 2016). As originally defined by Cavanagh and Lafortune (1980), we calculated the strike index as the distance from the heel to the center of pressure at impact relative to total foot length (thus the values range from 0 to 1). For the participants P0015 through P0032 the strike index values were not available.

### EMG Data

The muscle activity of the following 13 ipsilateral (right side) muscles was recorded (see Table 1 for details): *gluteus medius* (ME), *gluteus maximus* (MA), *tensor fasciæ latæ* (FL), *rectus femoris* (RF), *vastus medialis* (VM), *vastus lateralis* (VL), *semitendinosus* (ST), *biceps femoris* (long head, BF), *tibialis anterior* (TA), *peroneus longus* (PL), *gastrocnemius medialis* (GM), *gastrocnemius lateralis* (GL), and *soleus* (SO). We recorded two trials of 30 s for each participant. The EMG signals were high-pass filtered, then full-wave rectified and low-pass filtered (Santuz et al., 2017a) using a 4th order IIR Butterworth zero-phase filter with cut-off frequencies 50 Hz (high-pass) and 20 Hz (low-pass for the linear envelope) using R v3.5.1 (R Found. for Stat. Comp.). After filtering, any negative value was set to zero. Then, all the zero entries were set to the smallest non-zero value. The amplitude was normalized to the maximum activation recorded for each participant across both trials (Bizzi et al., 2008; Chvatal and Ting, 2013; Devarajan and Cheung, 2014; Santuz et al., 2018). Each gait cycle was then time-normalized to 200 points, assigning 100 points to the stance and 100 points to the swing phase (Santuz et al., 2017b, 2018). The reason for this choice was twofold. First, dividing the gait cycle into two macro-phases helps the reader understanding the temporal contribution of the different synergies, diversifying between stance and swing. Second, normalizing the duration of stance and swing to the same number of points for all participants (and for all the recorded gait cycles of each participant) is needed to make the interpretation of the results independent from the absolute duration of the gait events.

**Table 1:** Muscles considered for the extraction of muscle synergies (ipsilateral, right side of the body).

Upper leg	Gluteus medius^a^
	Gluteus maximus^b^
	Tensor fasciæ latæ^c^
	Rectus femoris
	Vastus medialis
	Vastus lateralis
	Semitendinosus
	Biceps femoris (long)
Lower leg	Tibialis anterior
	Peroneus longus
	Gastrocnemius medialis
	Gastrocnemius lateralis
	Soleus^d^

*Unless specified differently, the electrodes were positioned on the middle of muscle belly, along the main direction of the fibers. The specifications follow the SENIAM (Surface EMG for non-invasive assessment of muscles) recommendations. ^*a*^Middle of line between iliac crest and greater trochanter. ^*b*^Middle of line between sacral vertebræ and greater trochanter. ^*c*^Line from anterior spina iliaca superior to lateral femoral condyle in the proximal 1/6. ^*d*^At 2/3 of line between medial condyle of femur to medial malleolus.*

### Muscle Synergies Extraction

Muscle synergies were extracted through a custom script (Santuz et al., 2017b, 2018) (R v3.5.1, R Found. for Stat. Comp.) using the classical Gaussian NMF algorithm (Lee and Seung, 1999; Santuz et al., 2017a) from the first 30 gait cycles of each acquisition. The *m* = 13 time-dependent muscle activity vectors were grouped in an *m* × *n* matrix V (*n* = 30 gait cycles ^∗^ 200 points = 6000 points), factorized such that *V* ≈*V*_R_ = WH, where *V*_R_ represents the new reconstructed matrix, which approximates the original matrix *V*. The motor primitives (Dominici et al., 2011; Santuz et al., 2017a) matrix H contained the time-dependent coefficients of the factorization with dimensions *r* × *n*, where *r* represents the minimum number of synergies necessary to reconstruct the original signals (*V*). The motor modules (Gizzi et al., 2011; Santuz et al., 2017a) matrix *W* with dimensions *m* × *r*, contained the time-invariant muscle weightings, which describe the relative contribution of single muscles within a specific synergy (a weight was assigned to each muscle for every synergy). H and W described the synergies necessary to accomplish a movement. The update rules for H and W are presented in Equations (1) and (2).

$$\left\{ \begin{array}{l} H_{\text{i}+1\;}\;\;\;=H_{\text{i}}\frac{W_{\text{i}}^{\text{T}}V}{W_{\text{i}}^{\text{T}}W_{\text{i}}H_{\text{i}}}\;\;\;\;\;\;\;\;\;\;\;\;\;\;\;\;\;\;\left(1\right)\\ W_{\text{i}+1\;}\;\;\;=W_{\text{i}}\frac{V{\left(H_{\text{i}+1\;}\;\right)}^{\text{T}}}{W_{\text{i}}H_{\text{i}+1\;}{\left(H_{\text{i}+1\;}\right)}^{\text{T}}}\;\;\;\;\;\;\;\;\;\;\left(2\right) \end{array}\right.$$

The quality of reconstruction was assessed by measuring the coefficient of determination *R*^2^ between the original and the reconstructed data (*V* and *V*_R_, respectively). The limit of convergence was reached when a change in the calculated coefficient of determination *R*^2^ between *V* and *V*_R_ was smaller than the 0.01% in the last 20 iterations (Cheung et al., 2005; Santuz et al., 2017a), meaning that, with that amount of synergies, the signal could not be reconstructed any better. This operation was started by setting the number of synergies to 1. Then, it was repeated by increasing the number of synergies each time, until a maximum of 10 synergies. The number 10 was chosen to be lower than the number of muscles, since it would not make sense to extract a number of synergies equal to the number of measured EMG activities. The computation was repeated 10 times for each of the previous 10 steps, each time creating new randomized initial matrices H and W, in order to avoid local minima (D’Avella and Bizzi, 2005; Santuz et al., 2017a). The solution with the highest *R*^2^ was then selected for each of the 10 synergies. To choose the minimum number of synergies required to represent the original signals, the curve of *R*^2^ values versus synergies was fitted using a linear regression model, using all 10 synergies. The mean squared error (Cheung et al., 2005; Santuz et al., 2017a) between the curve and the linear interpolation was then calculated. Afterward, the first point in the *R*^2^-vs.-synergies curve was removed and the error between this new curve and its new linear interpolation was calculated. The operation was repeated until only two points were left on the curve or until the mean squared error fell below 10^-5^. This method searches for the most linear part of the *R*^2^-vs.-synergies curve and it is equivalent to stating that the reconstruction quality is not improving much when the curve becomes linear. With this approach, the need for setting a threshold to the reconstruction quality is avoided, giving space to the possibility that quality might not improve at the same rate when the same NMF algorithm is applied to different data.

The aforementioned procedure allowed us to extract fundamental and synergies from the raw EMG data. A fundamental synergy can be defined as an activation pattern whose motor primitive shows a single peak of activation (Santuz et al., 2017a). When two or more fundamental synergies are blended into one, a combined synergy is identified (Santuz et al., 2017a,b, 2018).

## Results

### Metadata

The file “participants_data.dat” is available at Zenodo (doi: 10.5281/zenodo.1254380) in ASCII and RData (R Found. for Stat. Comp.) format and contains:

• **Code**: the participant’s code
• **Sex**: the participant’s sex (M or F)
• **Speed**: the speed at which the recordings were conducted in [m/s]
• **Type**: gives information on whether the participant ran at their preferred (PR) or fixed (FX) speed
• **Age**: the participant’s age in years
• **Height**: the participant’s height in [cm]
• **Mass**: the participant’s body mass in [kg]
• **SI**: the strike index, dimensionless quantity defined as reported in the methods section, reported as the average value of all the steps recorded in both trials; values referred to the right foot.

### Gait Cycle Parameters

The files containing the gait cycle breakdown are available at Zenodo (doi: 10.5281/zenodo.1254380) in ASCII and RData (R Found. for Stat. Comp.) format. The files are structured as data frames with 30 rows (one for each gait cycle) and two columns. The first column contains the touchdown incremental times in seconds. The second column contains the duration of each stance phase in seconds. Each trial is saved both as a single ASCII file and as an element of a single R list. Trials are named like “CYCLE_TIMES_P0026_02,” where the characters “CYCLE_TIMES” indicate that the trial contains the gait cycle breakdown times, the characters “P0026” indicate the participant number (in this example the 26th) and the last two characters indicate the number of trial for that participant (either “01” for the first trial or “02” for the second). The average contact times were of 288 ± 42 ms, with an average swing time of 452 ± 45 ms at 163 ± 10 steps/min. The strike index of the right foot was on average 0.152 ± 0.195, with a maximum of 0.699 and a minimum of 0.011. In total, 82.5% of the participants had a strike index lower than 0.333 (rearfoot strike pattern).

### EMG Data

**FIGURE 1:**
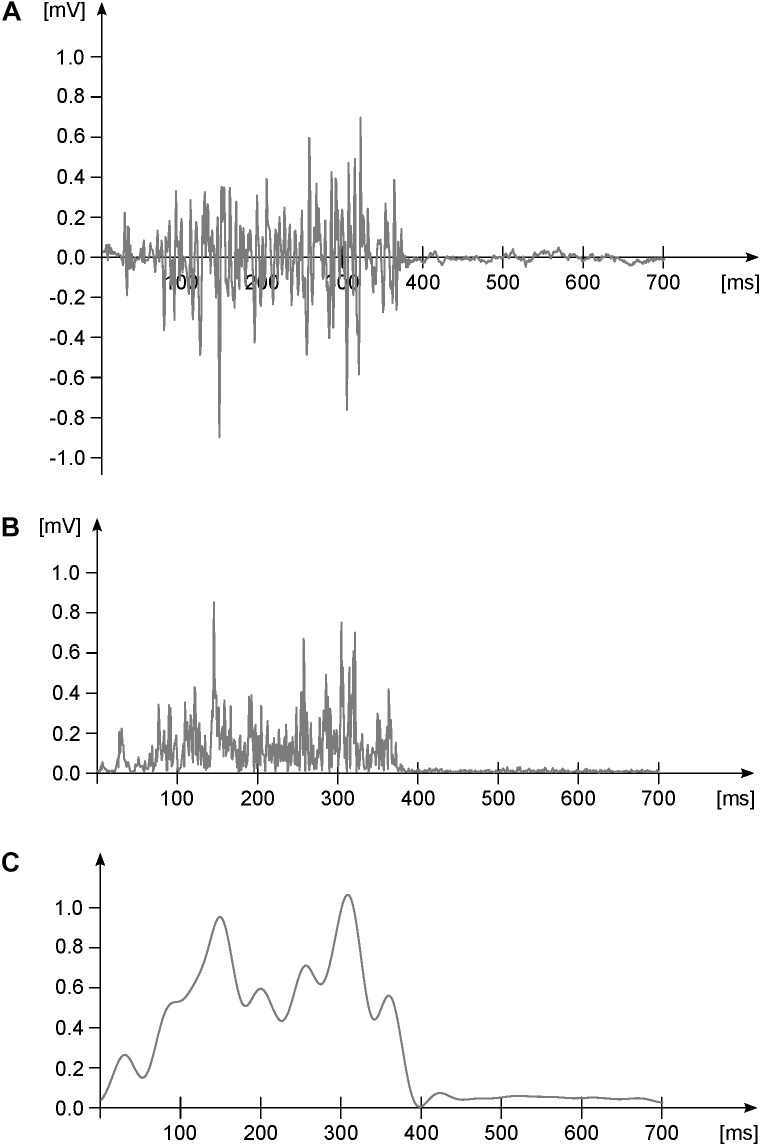
Exemplary EMG activity of one muscle during one gait cycle. **(A)** Raw data. **(B)** Raw data after high-pass filtering (4th order IIR Butterworth zero-phase filter, cut-off frequency 50 Hz) and full-wave rectification. Panel **(C)** rectified and high-pass filtered data after low-pass filtering (4th order IIR Butterworth zero-phase filter, cut-off frequency 20 Hz) and normalization to the maximum (dimensionless *y*-axis units). This last step is done for creating the linear envelope of the signal.

**FIGURE 2:**
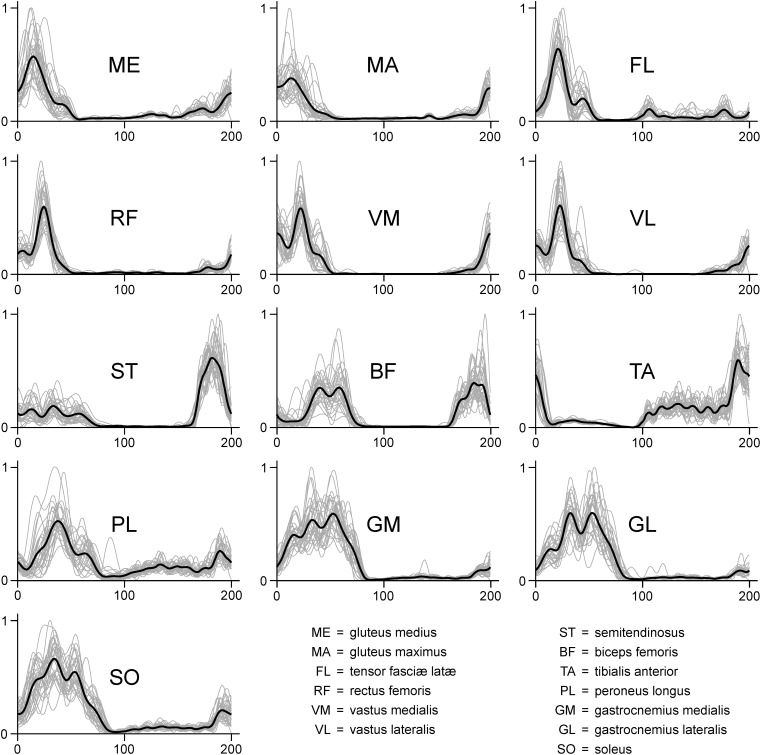
Exemplary EMG activity of the 13 recorded muscles recorded for one participant during one trial (treadmill running). Signals were high-pass filtered (4th order IIR Butterworth zero-phase filter, cut-off frequency 50 Hz), full-wave rectified, low-pass filtered (4th order IIR Butterworth zero-phase filter, cut-off frequency 20 Hz) and normalized to the maximum (dimensionless *y*-axis units). Since each gait cycle was time-normalized to 200 points, in each graph the first 100 points on the *x*-axis represent the stance phase, while the second 100 points represent the swing.

The files containing the raw, filtered and the normalized EMG data are available at Zenodo (doi: 10.5281/zenodo.1254380) in ASCII and RData (R Found. for Stat. Comp.) format. The raw EMG files are structured as data frames with 30000 rows (one for each recorded data point) and 14 columns. The first column contains the incremental time in seconds. The remaining thirteen columns contain the raw EMG data, named with muscle abbreviations that follow those reported in the “Materials and Methods” section of this paper. Each trial is saved both as a single ASCII file and as an element of a single R list. Figure 1 represents a typical filtering process for an EMG signal. In Figure 2 we report the EMG data acquired from one participant during one trial (cycles are superimposed and the average filtered signals are presented as well). Trials are named like “RAW_EMG_P0026_02,” where the characters “RAW_EMG” indicate that the trial contains raw EMG data, the characters “P0026” indicate the participant number (in this example the 26th) and the last two characters indicate the number of trial for that participant (either “01” for the first trial or “02” for the second). The filtered and time-normalized EMG data is named, following the same rules, like “FILT_EMG_P0026_02.”

### Muscle Synergies

The files containing the muscle synergies extracted from the filtered and normalized EMG data are available at Zenodo (doi: 10.5281/zenodo.1254380) in ASCII and RData (R Found. for Stat. Comp.) format. The muscle synergies files are divided in motor primitives and motor modules and are presented as direct output of the factorization and not in any functional order.

Motor primitives are data frames with a number of rows equal to the number of synergies (which might differ from trial to trial) and 6000 columns. The rows contain the time-dependent coefficients (motor primitives), one row for each synergy (named, e.g., “Syn1, Syn2, Syn3”, where “Syn” is the abbreviation for “synergy”). Each gait cycle contains 200 data points, 100 for the stance and 100 for the swing phase which, multiplied by the 30 recorded cycles, result in 6000 data points distributed in as many columns. Each set of motor primitives relative to one synergy is saved both as a single ASCII file and as an element of a single R list. Trials are named like “SYNS_H_P0026_02,” where the characters “SYNS_H” indicate that the trial contains motor primitive data, the characters “P0026” indicate the participant number (in this example the 26th) and the last two characters indicate the number of trial for that participant (either “01” for the first trial or “02” for the second).

Motor modules are data frames with 13 rows and a number of columns equal to the number of synergies (which might differ from trial to trial). The rows, named with muscle abbreviations that follow those reported in the methods section of this paper, contain the time-independent coefficients (motor modules), one for each synergy and for each muscle. Each set of motor modules relative to one synergy is saved both as a single ASCII file and as an element of a single R list. Trials are named like “SYNS_W_P0026_02,” where the characters “SYNS_W” indicate that the trial contains motor module data, the characters “P0026” indicate the participant number (in this example the 26th) and the last two characters indicate the number of trial for that participant (either “01” for the first trial or “02” for the second).

**FIGURE 3:**
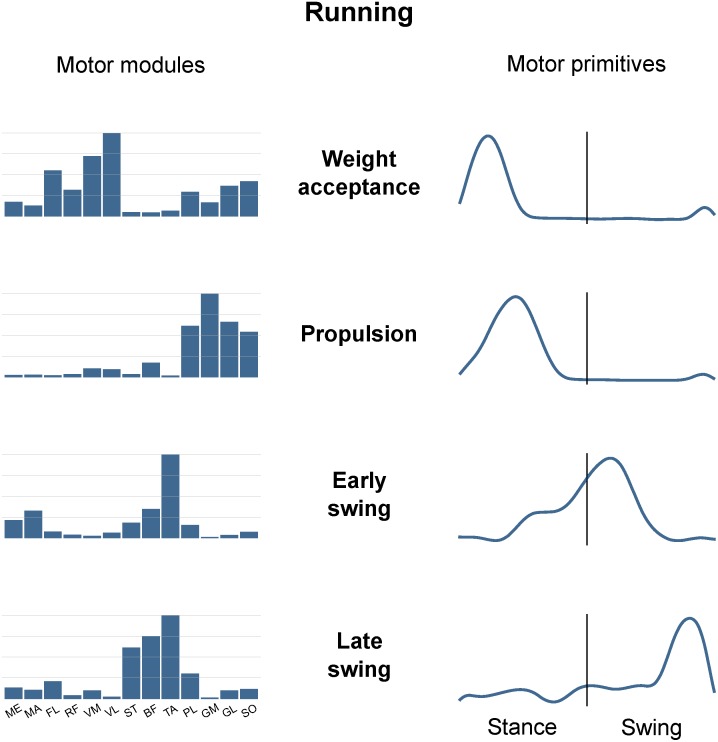
Exemplary motor modules and motor primitives of the four fundamental synergies for human running (one trial). The motor modules are presented on a normalized y-axis base. For the motor primitives, the x-axis full scale represents the averaged gait cycle (with stance and swing normalized to the same amount of points and divided by a vertical line) and the *y*-axis the normalized amplitude. Muscle abbreviations: ME, gluteus medius; MA, gluteus maximus; FL, tensor fasci lat; RF, rectus femoris; VM, vastus medialis; VL, vastus lateralis; ST, semitendinosus; BF, biceps femoris; TA, tibialis anterior; PL, peroneus longus; GM, gastrocnemius medialis; GL, gastrocnemius lateralis; SO, soleus.

Figure 3 is an example of how muscle synergies can be graphically represented. The recorded muscle activations can be approximated by the linear combination of motor modules and motor primitives. Since they are time-invariant coefficients, motor modules are usually represented with bar graphs. On the contrary, motor primitives describe the evolution over time of the basic activation patterns and are therefore better represented with time-dependent curves. When multiplying and summing synergy-by-synergy the elements of the two matrices W (motor modules) and H (motor primitives), it is possible to reconstruct the original set of EMG data. For instance, it is possible to notice from Figure 3 that the muscle PL, GM, GL, and SO are the major contributors to the second synergy, named “Propulsion.” In fact, these ankle plantar flexors are important during the push-off in running, a phase that chronologically succeeds the weight acceptance (first synergy) and precedes the early swing (third synergy). The chronological order of synergies can be seen in the motor primitives, the fundamental activation patterns that describe the evolution over time of those commands which are common to differently functional groups of muscles (e.g., the plantar flexors in the second synergy).

The minimum number of synergies necessary to sufficiently describe the measured EMG activity during running was 4.7 ± 0.7. Excluding the combined synergies, four fundamental activation patterns could be identified (Figure 3). The four fundamental synergies were associated with temporally different phases of the gait cycle. The first synergy functionally referred to the body weight acceptance, with a major involvement of knee extensors and glutei. The second synergy described the propulsion phase, to which the plantar flexors mainly contributed. The third synergy identified the early swing, showing the involvement of foot dorsiflexors. The fourth and last synergy reflected the late swing and the landing preparation, highlighting the relevant influence of knee flexors and foot dorsiflexors.

### Code

All the code used for the preprocessing of EMG data and the extraction of muscle synergies is available at Zenodo (doi: 10.5281/zenodo.1254380) in R (R Found. for Stat. Comp.) format. Explanatory comments are profusely present throughout the scripts (“SYNS.R,” which is the main script and “fun_synsNMFn.R,” which contains the NMF function).

## Discussion

With this study, we make available a large data set of lower-limb EMG activity recorded during human running. Data was acquired from 13 ipsilateral lower limb muscles in 135 young and healthy individuals. An exemplary script, which can be used to pre-process and factorize the EMG data via NMF, is also part of the data set. A metadata file contains the relevant demographic and anthropometric data of the participants, together with important information regarding the experimental conditions and the general guidelines to interpret muscle synergy data.

The etymology of the word “synergy” is nested in the Greek language. Literally, synergy means “working together” (συνεργóς). The idea that some synergistic neural components of movement exist was already suggested by Sherrington (1906) at the beginning of the 20th century. In his famous “The integrative action of the nervous system,” Sherrington (1906) wrote “The stimulation […] excites reflexly through the central organ an effect in the skeletal musculature which is co-ordinate and synergic.” Yet, Sherrington (1906) took some distance from the concept of a functional organization of the motor spinal root, arguing that “the collection of fibers in a spinal motor root is not a functional collection in the sense that it is representative of any co-ordination.” Bernstein (1967) published his “The co-ordination and regulation of movements,” a book that became a milestone in the history of muscle synergies. For the first time, Bernstein (1967) formally described the so-called “degrees of freedom problem,” stating that “the basic difficulties for co-ordination consist precisely in the extreme abundance of degrees of freedom, with which the [CNS] […] is not at first in a position to deal.” This concept of motor abundance is still one of the supporting pillars of modern motor control and laid the foundation of the muscle synergies idea. In the past two decades, the scientific publications embracing the concept of muscle synergies have been flourishing and exponentially increasing in number. Even if the consensus on factorization techniques, data conditioning and interpretation is not unanimous, it is well accepted that human locomotion can be described with a small number of synergies. When analyzing the EMG activities of lower limb’s muscles (Santuz et al., 2017b, 2018), this number is usually equal to 4 or 5. A synergy might add when considering the upper body (Cappellini et al., 2006; Santuz et al., 2017a).

There are several examples of studies employing factorization of EMG activity to study human locomotion. For several reasons, the most widespread locomotion type that has been studied is walking (Ivanenko et al., 2004; Cappellini et al., 2006; Courtine et al., 2006; Clark et al., 2010; McGowan et al., 2010; Dominici et al., 2011; Allen and Neptune, 2012; Bolton and Misiaszek, 2012; Chvatal and Ting, 2012, 2013; Lacquaniti et al., 2012; Oliveira et al., 2012; Rodriguez et al., 2013; Barroso et al., 2014; Maclellan et al., 2014; Routson et al., 2014; Coscia et al., 2015; Gonzalez-Vargas et al., 2015; Hagio et al., 2015; Licence et al., 2015; Martino et al., 2015; Nazifi et al., 2015; Tang et al., 2015; Buurke et al., 2016; Gui and Zhang, 2016; Kim et al., 2016; Lencioni et al., 2016; Meyer et al., 2016; Pérez-Nombela et al., 2016; Yokoyama et al., 2016; Allen et al., 2017; Janshen et al., 2017; Santuz et al., 2017a; Shuman et al., 2017; Saito et al., 2018). Due to the easiness of examining this slow-speed type of locomotion, it is not a surprise that the majority of studies use walking as the main object of investigation. Also, it is clear that, contrarily to other locomotion types such as running, walking can be easily performed by patients, children and elderly and this feature notably extends the basin of potential participants. Nonetheless, running has been receiving increasing attention (Cappellini et al., 2006; Lacquaniti et al., 2012; Hagio et al., 2015; Yokoyama et al., 2016; Nishida et al., 2017; Santuz et al., 2017a,b, 2018) as well. This might be partially due to the growing popularity of distance running as a recreational sport activity over the last three decades (Burfoot, 2007). Another reason to choose running over walking (or to study both conditions within the same experimental setup) is that, due to the different absolute and relative length of the stance and swing phases, different control mechanisms are likely to be used by the CNS (Biewener and Daley, 2007; Santuz et al., 2018). Concerning this last matter, though, the field is still much open to new ideas, insights and exciting findings (Santuz et al., 2018). Unavoidably, the links between locomotion velocity and modular organization have been investigated as well (Ivanenko et al., 2004; Cappellini et al., 2006; Routson et al., 2014; Coscia et al., 2015; Gonzalez-Vargas et al., 2015; Hagio et al., 2015; Buurke et al., 2016; Gui and Zhang, 2016; Yokoyama et al., 2016). However, results are often contradictory and the reasons have not yet been clarified. Whether for computational or neurophysiological reasons, some studies found consistency in the recruitment of the same motor primitives and/or modules across varying velocities (Ivanenko et al., 2004; Cappellini et al., 2006; Routson et al., 2014; Buurke et al., 2016; Gui and Zhang, 2016), while others found walking-, running-, and/or velocity-specific sets of motor primitives and/or modules (Cappellini et al., 2006; Routson et al., 2014; Coscia et al., 2015; Gonzalez-Vargas et al., 2015; Yokoyama et al., 2016). The role of muscle synergies for locomotion in pathology has been a focus of a few groups in recent years (Latash and Anson, 2006; Clark et al., 2010; Giszter and Hart, 2013; Rodriguez et al., 2013; Routson et al., 2014; Coscia et al., 2015; Tang et al., 2015; Falaki et al., 2016; Lencioni et al., 2016; Meyer et al., 2016; Pérez-Nombela et al., 2016; Shuman et al., 2016, 2017; Wenger et al., 2016; Allen et al., 2017; Banks et al., 2017). Given the simplification in presenting the data due to the dimensionality reduction, it is appealing to think to a possible clinical application of the method. There have been comparisons between healthy and Parkinson’s disease (Rodriguez et al., 2013; Falaki et al., 2016; Allen et al., 2017), multiple sclerosis patients (Lencioni et al., 2016), spinal cord injury (Giszter and Hart, 2013; Pérez-Nombela et al., 2016; Wenger et al., 2016), cerebral palsy (Li et al., 2013; Steele et al., 2015; Tang et al., 2015; Shuman et al., 2016, 2017), and post-stroke (Clark et al., 2010; Routson et al., 2014; Coscia et al., 2015; Meyer et al., 2016; Banks et al., 2017) patients. However, as for the studies on the influence of velocity on the modular organization of motion, also in pathology studies results are often difficult to interpret and require careful analysis. The study of the modular organization of locomotion in unsteady conditions has as well started to meet the interest of some research groups (Chvatal and Ting, 2012; Oliveira et al., 2012; Licence et al., 2015; Martino et al., 2015; Nazifi et al., 2015; Santuz et al., 2018), highlighting the importance of extending the controlled laboratory conditions to daily life.

The big, open access data set we present in this study, serves a threefold purpose. First, it increases the representative power of the data which is commonly obtainable with a standard experimental setup. Usually, due to experimental or design constraints, 5 to 20 individuals are recruited for each measurement campaign (Cappellini et al., 2006; Hagio et al., 2015; Yokoyama et al., 2016; Nishida et al., 2017; Santuz et al., 2017a,b, 2018). The choice is often dictated by the limited time available, difficulties in recruiting volunteers, budget limits, etc. With this publication, we make 135 (at the time of publication) young and healthy participants’ data freely available and ready for numerical analysis. Our data can establish a baseline for those studies that aim to investigate, amongst others, different populations (such as elderly, children, patients, etc.) or conditions (walking, perturbed locomotion, etc.). Therefore, compared to a standard setup, the increased number of participants included in our study can be a prime source for broadening the representation of human motor control. While small samples might fail to capture the variety of population, the 135 proposed samples provide a preferential lane toward a more comprehensive description of the modular control of movement.

Second, the data could be used for many different scientific purposes in several research fields. For instance, both EMG and synergies data might be employed for the development of more advanced musculoskeletal models (Lai et al., 2014). Another possible application would be improving the control of active exoskeletons or robots for aiding or substituting human movement (Lai et al., 2014). The torques needed to generate a certain movement can be computed, but the complexity of motion equations dramatically increases with the number of degrees of freedom (D’Avella, 2016). Thus, synergies might be an effective way to store approximate yet sufficient information to build motor commands (D’Avella, 2016). This big data set might help scientists to transfer the knowledge coming from data acquired *in vivo* to *in silico* controls, providing a benchmark for what can be expected from artificial movement control.

Third, the data set could be used by other members of the scientific community interested in improving the existing or creating new muscle synergies extraction methods (Févotte et al., 2009; Devarajan and Cheung, 2014; Santuz et al., 2017a; Shuman et al., 2017; Kieliba et al., 2018; Soomro et al., 2018). This would greatly improve comparability across groups working in the field. For instance, several update rules have been and are continuously proposed for data factorization via NMF in a constant effort to improve their computational performance in terms of reconstruction capabilities and speed (Févotte et al., 2009; Devarajan and Cheung, 2014; Santuz et al., 2017a). However, to date, the classical Gaussian approach is the most used for EMG decomposition (Cappellini et al., 2006; Dominici et al., 2011; Santuz et al., 2017b). Also the choice of the minimum number of synergies necessary to sufficiently reconstruct the original signal is still matter of debate. Answering the question “how good is good enough?” has often led to an oversimplification of the issue, with many publications solving the problem by setting an arbitrary threshold on the *R*^2^ values (Chvatal and Ting, 2013; Tang et al., 2015; Nishida et al., 2017). Moreover, some studies already investigated the influence of EMG preprocessing on muscle synergies (Santuz et al., 2017a; Shuman et al., 2017; Kieliba et al., 2018). Our data set provides a starting point for this kind of methodological studies. Last but not least, the educational potential of this data could be used to train students at all levels and from many different disciplines, from sport science, to medicine, from engineering, to mathematics and so forth.

It must be taken into account, however, that this data set has some limitations. First of all, it only includes data from young and healthy individuals. Thus, the data cannot be directly transferred to the study of children, adolescents, or elderly. Moreover, the muscles included in the recordings are limited to the lower limb. For extended considerations on the contribution of the upper body to the modular organization of running, more muscles should be included (Santuz et al., 2017a). Then, data was not recorded at the same speed for all participants, even if the average speed was close to the population’s preferred (Santuz et al., 2016). Lastly, even though overground and treadmill running have been shown to share similar modular organization (Oliveira et al., 2016), this data set does only provide treadmill data.

## Author Contributions

AS, AE, LJ, FM, SB, VB, and AA contributed to conceptualization, writing the review, and editing. AS, LJ, and AA contributed to methodology. AS, AE, LJ, FM, and SB contributed to investigation. AS contributed to formal analysis and visualization. AS and AA wrote the original draft. VB and AA contributed to supervision.

## Conflict of Interest Statement

The authors declare that the research was conducted in the absence of any commercial or financial relationships that could be construed as a potential conflict of interest.
